# Predictors for symptomatic intracranial hemorrhage after intravenous thrombolysis with acute ischemic stroke within 6 h in northern China: a multicenter, retrospective study

**DOI:** 10.1186/s12883-021-02534-9

**Published:** 2022-01-03

**Authors:** Yuan Xue, Shan Li, Yuanyuan Xiang, Ziran Wang, Fengyun Wang, Yuanying Yu, Peng Yan, Xiaohui Liu, Qinjian Sun, Yifeng Du, Jifeng Li

**Affiliations:** 1 Department of Neurology, Shandong Provincial Hospital Affiliated to Shandong First Medical University, No. 324 Jingwu Weiqi Road, Jinan, Shandong 250021 PR China; 2 Department of Emergency, Linyi People’s Hospital Affiliated to Shandong University, Lin yi, Shandong China; 3 Department of Neurology, Liaocheng Brain Hospital, Liaocheng, Shandong China; 4 Department of Neurology, Haiyang People’s Hospital, Haiyang, Shandong China

**Keywords:** Stroke, Intravenous thrombolysis, Intracranial hemorrhages, Predictor, Risk factors

## Abstract

**Background and purpose:**

This study assessed the predictive factors for symptomatic intracranial hemorrhage (sICH) in patients with acute ischemic stroke (AIS) after receiving intravenous thrombolysis (IVT) within 6 h in northern China.

**Methods:**

We retrospectively analyzed ischemic stroke patients who were treated with IVT between November 2016 and December 2018 in 19 hospitals in Shandong Province, China. Potential predictors of sICH were investigated using univariate and multivariate analyses.

**Results:**

Of the 1293 enrolled patients (845 men, aged 62 ± 11 years), 33 (2.6%) developed sICH. The patients with sICH had increased coronary heart disease (36.4% vs. 13.7%, *P* = 0.001), more severe stroke (mean National Institutes of Health Stroke Scale [NIHSS] score on admission of 14 vs.7, *P* < 0.001), longer door-to-needle time [DNT] (66 min vs. 50 min, *P* < 0.001), higher blood glucose on admission, higher white blood cell counts (9000/mm^3^ vs. 7950/mm^3^, *P* = 0.004) and higher neutrophils ratios (73.4% vs. 67.2%, *P* = 0.006) et al. According to the results of multivariate analysis, the frequency of sICH was independently associated with the NIHSS score (OR = 3.38; 95%CI [1.50–7.63]; *P* = 0.003), DNT (OR = 4.52; 95%CI [1.69–12.12]; *P* = 0.003), and white blood cell count (OR = 3.59; 95%CI [1.50–8.61]; *P* = 0.004).

When these three predictive factors were aggregated, compared with participants without any factors, the multi-adjusted odds ratios (95% confidence intervals) of sICH for persons concurrently having one, two or three of these factors were 2.28 (0.25–20.74), 15.37 (1.96–120.90) and 29.05 (3.13–270.11), respectively (P for linear trend < 0.001), compared with participants without any factors.

**Conclusion:**

NIHSS scores higher than 10 on admission, a DNT > 50 min, and a white blood cell count ≥9000/mm^3^ were independent risk factors for sICH in Chinese patients within 6 h after IVT for AIS.

## Introduction

Intravenous thrombolysis (IVT) treatment is an effective therapy for acute ischemic stroke (AIS), which includes providing recombinant tissue plasminogen activator (rt-PA) to patients within 4.5 h [1] and urokinase (UK) within 6 h in China [2]. However, symptomatic intracranial hemorrhage (sICH) is one of the most feared complications after thrombolytic therapy [3, 4], and is associated with neurological deterioration and poor outcome. Thus, identifying the predictors of sICH in patients receiving IVT is crucial.

SICH has been defined slightly differently among previous studies; the incidence rate of sICH varies between 2.0 and 7.2% worldwide [5–8], and the incidence of sICH in Asia is higher [9] or equivalent [8] to that in Western countries, according to mSITS-MOST (the modified version of Safe Implementation of Thrombolysis in Stroke-Monitoring Study) criteria. Different factors, such as a higher National Institutes of Health Stroke Scale (NIHSS) score, advanced age, elevated serum glucose levels and cardioembolism, have been suggested as predictors of sICH after IVT within 4.5 h of AIS in China (TIMS-China) study [5].

Given that UK is much cheaper and may have an apparent longer therapeutic time window than rt-PA, Chinese guidelines recommend UK for use within 6 h of the onset of AIS as an alternative to rt-PA [10]. However, to our knowledge, the incidence and predictors of sICH in the Chinese patients with AIS treated with rt-PA or UK within 6 h remain unclear, and it is worthwhile to explore the risk factors for sICH based on multicenter retrospective study data in northern China. In summary, identifying the risk factors for sICH has important practical significance for communicating with patients and their relatives and taking efficacious measures to prevent sICH.

## Materials and methods

### Study design

We retrospectively analyzed ischemic stroke patients who received IVT, including rt-PA and urokinase, within 6 h of stroke onset at 19 hospitals in Shandong Province, China, between November 2016 and December 2018. Inclusion and exclusion criteria for IVT were mainly adopted from the National Institute of Neurological Disorders and Stroke protocol and the protocol of the Chinese Guidelines for the Management of Stroke. Patients treated with endovascular treatment after IVT, patients with known tumors, inflammation, treatment administration > 6 h after symptom onset, no follow-up brain imaging after treatment administration and unavailable blood samples were excluded from this study. The selection of different thrombolytic doses and treatment strategies was completely determined by the institution or the treating physician and was not standardized. We assessed stroke severity by the baseline NIHSS score, and the presumed cause of ischemic stroke with the international Trial of ORG 10172 in Acute Stroke Treatment (TOAST) classification [11]. All TOAST classification assignments were further verified by an experienced senior neurologist (Jifeng Li).

Data on patient demographics, clinical characteristics and the use of thrombolysis medications were collected from patients’ charts by neurologists from the participating hospitals. Potential risk factors included age, sex, hypertension, diabetes mellitus, dyslipidemia, coronary heart disease, atrial fibrillation, door-to-needle time (DNT) (minutes), Onset-to-needle time (ONT) (minutes), NIHSS scores on admission, systolic blood pressure before thrombolysis, diastolic blood pressure before thrombolysis, smoking status, alcohol consumption, blood glucose on admission, antiplatelet treatment pre-IVT, anticoagulant treatment pre-IVT, serum white blood cell (WBC) count, neutrophil-WBC ratio (neutrophil ratio), platelet count, low-density lipoprotein cholesterol (LDL-C) level on admission, rt-PA dose (mg/kg body weight), urokinase dose (million units), and stroke subtype.

The study was approved by the Ethical Standards Committee on Human Experimentation at Shandong Provincial Hospital Affiliated to Shandong First Medical University. A signed consent form was obtained from all participants. The study was conducted in accordance with the principles for medical research involving human subjects expressed in the Declaration of Helsinki.

### Outcome measures

The primary outcome was the incidence of symptomatic intracranial hemorrhage (sICH) between 0 and 36 h after IVT. SICH was based on parenchymal hemorrhage with deterioration using the National Institutes of Health Stroke Scale score of ≥4 points or death (the modified Safe Implementation of Thrombolysis in Stroke- Monitoring Study [mSITS-MOST]) [12].

### 1.3 Statistical analysis

For categorical variables, the chi-square (χ2) test or Fisher’s exact test was presented as percentages. For continuous variables, the t-test or the Mann–Whitney U-test was presented as the means ± SD or median (interquartile range). Variables with *P* < 0.05 in the univariate analysis were entered into the multivariate logistic regression analysis. Receiver operating characteristic curve analysis was performed for continuous variables with statistical significance in the logistic regression analysis to analyze the predictability of sICH. A multiple logistic regression model was used to estimate the odds ratios (ORs) and 95% confidence intervals (CIs) of sICH associated with individual risk factors and their load, which was assessed by counting the number of risk factors that were significantly related to an increased odds ratio of sICH (*P* < 0.05). We reported the results from two models: Model 1 was unadjusted, and Model 2 was adjusted for coronary heart disease, neutrophil ratio, and stroke subtype. A two-sided *p* value of < 0.05 was considered statistically significant. All analyses were performed with the SPSS 22.0 software.

## Results

From November 2016 to December 2018, 1541 patients with AIS from 19 hospitals participated in this retrospective study. Among them, 22 patients who received IVT > 6 h after stroke onset were excluded, 56 patients who did not undergo follow-up brain CT after recanalization were excluded, and another 2 patients were excluded because of a lack of rt-PA dose record data. Furthermore, 168 patients who received endovascular treatment were also excluded. Thus, a total of 1293 patients with onset-to-needle time < 6 h met our study criteria. Among them, 33 (2.6%) experienced mSITS-MOST-defined sICH.

The demographic and baseline characteristics of the patients with sICH are summarized in Table 1. Compared with patients without sICH, sICH patients had more severe stroke (defined by higher NIHSS scores) on admission, longer DNT, increased history of coronary heart disease, higher blood glucose on admission, higher neutrophil ratio and white blood cell counts, higher rt-PA doses (0.9 mg/kg vs. 0.6 mg/kg or 50 mg/person), and increased rates of cardioembolism.

**Table 1:** Demographic and baseline characteristics of study participants by sICH per mSITS-MOST criteria

Variables	sICH (*n* = 33)	No sICH (*n* = 1260)	*P*-value
Age (years)	65 (61–72)	64 (55–71)	0.153^a^
Sex, %			
Male	21 (63.6)	824 (65.4)	0.854
Female	12 (36.4)	436 (34.6)	
DNT (min)	66 (55–95)	50 (35–69)	< 0.001^a^
Onset-to-needle time (ONT) (min)	182 (143–215)	171 (140–195)	0.267^a^
Baseline NIHSS	14 (10–18)	7 (4–12)	< 0.001^a^
Hypertension, n (%)	19 (57.6)	734 (58.3)	1.000
Diabetes mellitus, n (%)	7 (21.2)	198 (15.7)	0.466
Dyslipidemia, n (%)	1 (3.0)	26 (2.1)	0.506
Coronary heart disease, n (%)	12 (36.4)	172 (13.7)	0.001
Atrial fibrillation, n (%)	6 (18.2)	89 (7.1)	0.060
Systolic blood pressure before thrombolysis, mm Hg	151 (145.5–155)	151 (140–164)	0.610^a^
Diastolic blood pressure before thrombolysis, mm Hg	86 (83–95)	86 (80–94)	0.210^a^
Smoking, n (%)	11 (33.3)	445 (35.3)	0.856
Alcohol consumption, n (%)	11 (33.3)	397 (31.5)	0.850
Blood glucose on admission (mmol/L)	9.1 (5.9–12.3)	8.3 (5.2–11.4)	0.045^a^
Antiplatelet treatment pre-IVT, n (%)	7 (21.2)	146 (11.6)	0.178
Anticoagulant treatment pre-IVT, n (%)	1 (3.0)	19 (1.5)	0.244
Neutrophils ratio (%)^b^	73.4 (63.2–81.4)	67.2 (61.0–73.0)	0.006^a^
Platelet count (× 1000/mm^3^)	195.0 (170.3–231.5)	223.3 (192.0–248.0)	0.059^a^
White blood cell (×1000/mm^3^)	9.0 (7.3–10.9)	7.95 (6.03–8.40)	0.004^a^
LDL-C, mmol/L	2.89 (2.38–2.99)	2.97 (2.53–3.33)	0.155^a^
Use of thrombolysis medications, n (%)			
rtPA	26 (78.8)	1070 (84.9)	0.227
urokinase	7 (21.2)	190 (15.1)	
Dose of rt-PA			
No use	7 (21.2)	190 (15.1)	< 0.001
0.9 mg/kg	26 (78.8)	602 (47.8)	
0.6 mg/kg	0 (0)	22 (1.7)	
50 mg/person	0 (0)	446 (35.4)	
Dose of urokinase, n (%)			
No use	26 (78.8)	1070 (84.9)	0.281
1 million units	6 (18.2)	173 (13.7)	
1.5 million units	1 (3.0)	17 (1.3)	
Stroke subtype, %			
Atherothrombotic	18 (54.5)	972 (77.1)	< 0.001
Small vessel disease	3 (9.1)	205 (16.3)	
Cardioembolism	10 (30.3)	60 (4.8)	
Unknown or other etiology	2 (6.1)	23 (1.8)	

^a^Statistical significance, according to a Mann-Whitney U-test

^b^Proportion (%) of neutrophils in the WBC

Fisher’s exact test: atrial fibrillation, coronary heart disease, diabetes mellitus, dyslipidemia, use of thrombolysis medications, dose of rt-PA, dose of urokinase, and stroke subtype.

We categorized the data into 2 categories based on the standard cut-points or previous studies. According to univariable analysis, the parameters selected for further multivariable analysis (*P* < 0.05) were: DNT > 50 min, prior coronary heart disease, baseline NIHSS scores > 10, neutrophil ratio prior to thrombolysis ≥71.2%, white blood cell count prior to thrombolysis ≥9000/mm^3^, and stroke subtype (Table 2).

**Table 2:** Results of univariable logistic regression analysis of sICH risk factors (ordinal odds ratios)

Item	OR (95%CI)	*P*-value
DNT (min)		
0–50	1	
>50	6.259 (2.401–16.313)	< 0.001
Coronary heart disease	3.615 (1.747–7.480)	0.001
Blood glucose on admission ≥9 mmol/L	1.938 (0.834–4.501)	0.124
Neutrophils ratio ≥ 71.2(%)	3.227 (1.601–6.505)	0.001
White blood cell ≥9000/mm^3^	4.187 (2.087–8.402)	< 0.001
Dose of rt-PA		
No use	1	
0.9 mg/kg	1.172 (0.501–2.744)	0.714
0.6 mg/kg	NA	0.998
50 mg/person	NA	0.992
Stroke subtype		
Atherothrombotic	1	
Small vessel disease	0.790 (0.231–2.708)	0.708
Cardioembolic	7.493 (3.125–17.969)	< 0.001
Unknown or other etiology	4.696 (1.029–21.432)	0.006
Baseline NIHSS		
0–10	1	
> 10	5.772 (2.720–12.250)	< 0.001
Discharge mRS (0–2)	0.142 (0.070–0.287)	< 0.001
90-day mRS (0–2)	0.166 (0.069–0.400)	< 0.001

Independent factors associated with sICH were analyzed using the multivariate logistic regression. The highest odds ratios were DNT >50 min (vs. ≤ 50) (OR, 4.52; 95% CI, 1.69–12.12; *P* = 0.003), the NIHSS scores on admission > 10 (vs. ≤ 10) (OR, 3.38; 95% CI, 1.50–7.63; P = 0.003), and white blood cell count ≥9000/mm^3^ (OR, 3.59; 95% CI, 1.50–8.61; *P* = 0.004) (Table 3).

**Table 3:** Results of multivariable logistic regression analysis of sICH risk factors (ordinal odds ratios)

Item	OR (95%CI)	*P*-value
DNT (min)		
0–50	1	
>50	4.520 (1.686–12.123)	0.003
Coronary heart disease	1.644 (0.678–3.985)	0.271
Neutrophils ratio ≥ 71.2(%)	1.135 (0.480–2.683)	0.774
White blood cell ≥9000/mm^3^	3.593 (1.499–8.612)	0.004
Stroke subtype		
Atherothrombotic	1	
Small vessel disease	1.001 (0.278–3.608)	0.998
Cardioembolic	2.289 (0.934–5.534)	0.058
Unknown or other etiology	2.433 (0.897–6.598)	0.081
Baseline NIHSS		
0–10	1	
> 10	3.383 (1.500–7.632)	0.003

When these three predictive factors were aggregated, the multi- adjusted OR (95% confidence interval) of having sICH was increased significantly with an increasing number of concurrent predictive factors (P for linear trend < 0.001) (Table 4).

**Table 4:** Association of risk factors load with symptomatic intracranial hemorrhage (sICH)

No. of risk factors ^a^	No. of subjects	No. of sICH cases	Odds ratio (95% confidence interval)	
			Model 1^b^	Model 2^c^
Continuous variable (range 0–3)	1293	33	4.29 (2.73–6.74) ^‡^	3.76 (2.19–6.44) ^‡^
Categorical variable				
0	435	1	1.00 (Reference)	1.00 (Reference)
1	511	4	3.42 (0.38–30.74)	2.28 (0.25–20.74)
2	272	19	28.98 (3.83–219.06) ^†^	15.37 (1.96–120.90) ^†^
3	58	9	69.28 (8.49–565.39) ^‡^	29.05 (3.13–270.11) ^†^
P for trend			< 0.001	< 0.001

^a^Three risk factors were aggregated including NIHSS on admission higher than 10, DNT >50 min, and white blood cell ≥9000/mm^3^

^b^Model 1: Unadjusted

^c^Model 2: Adjusted for coronary heart disease, neutrophils ratio, and stroke subtype

^†^*P* < 0.05

^‡^*P* < 0.001

## Discussion

In this multicenter retrospective study, we observed that sICH occurred in 2.6% of patients by the mSITS-MOST definition in our research, which is comparable to incidence reported in the TIMS-China trial (2.0%) [5]. However, patients who received only intravenous rt-PA within 4.5 h of AIS onset were recruited for the TIMS-China trial. Furthermore, our report showed that higher NIHSS score, higher white blood cell count on admission, and delayed recanalization treatment were independently associated with sICH after IVT. Our study seems to be the first multicenter retrospective study conducted in northern China to investigate the predictors of sICH with AIS patients treated with rt-PA or UK within 6 h, which may better reflect real-world practices.

Compared with patients without sICH, sICH patients had higher blood glucose on admission in our research (mean 9.1 versus 8.3 mmol/L, *P* = 0.045) (Table 1). In addition, we found no statistical association between admission blood glucose and increasing the risk of sICH post IVT according to mSITS- MOST criteria (OR = 1.938; *P* = 0.124) (Table 2), a finding reported in prior studies [13, 14]. However, in previous studies [5, 15], elevated baseline glucose level was shown to be a risk factor for sICH in acute ischemic stroke patients treated with IV rt-PA, in which the sICH was diagnosed based on the European Cooperative Acute Stroke Study II (ECASS-II) definition. Certainly, the mechanism behind this phenomenon requires further research.

High white blood cell (WBC) counts are known to be involved in the inflammatory process of AIS [16]. A study by Tiainen et al. reported that higher WBC counts at admission were significantly associated with sICH in AIS patients treated with IVT [17], and similar findings were obtained in our research. In contrast, another study indicated that elevated WBC counts failed to independently predict sICH [18]. In our research, the best predictor of sICH was the WBC count in blood tests, and a value ≥9000/mm^3^ indicated a 3.6-fold increased risk for sICH. The different results of previous studies may have been caused by the different times of blood sampling due to the dynamic changes in WBC counts after stroke [19]. The mechanism behind the association between elevated WBC counts and sICH is not completely understood, which may be partly because AIS causes WBCs to migrate into the brain, where they can cause brain edema and injury by initiating inflammatory cascade reactions and releasing inflammatory cytokines [20].

Among the clinical risk factors, DNT and NIHSS scores were independently associated with sICH in our study, and these results are consistent with those of previous studies [5, 13]. The elapsed time from hospital admission to the thrombolytic bolus was defined as door-to-needle time (DNT). The benefit of IVT for patients with AIS is time-dependent. The clinical benefit from IVT declines rapidly (time is brain), and every minute counts [21]. In our report, DNT turned out to be the most reliable predictor of sICH among the other parameters assessed, which revealed that patients with a DNT > 50 min had a 4.5-times higher risk of developing sICH than those with a DNT ≤ 50 min, and the difference was statistically significant (*P* = 0.003) when adjusted for multivariate analysis.

The National Institutes of Health Stroke Scale (NIHSS) score is a tool used to objectively quantify stroke impairment of the stroke and is the most commonly used stroke outcome scale [22]. Higher NIHSS scores are generally associated with the more severe ischemic stroke, which is reflected by large areas of injured blood vessels that are prone to bleeding after IVT. In our research, a higher initial NIHSS score on admission increased the risk of sICH, which was similar to the findings of previous reports [23, 24]. We found that patients with NIHSS scores > 10 had a 3.4-times higher risk of sICH (using NIHSS scores ≤10 as reference). If a patient that had an acute stroke and receiving IVT has an NIHSS score > 10, the physician must be cautious and should check the patient’s white blood cell count. It is worth noting that the NIHSS-assessed stroke severity was milder in the current study than in previous studies, which may explain why the sICH incidence (2.6%) was lower than that in previous reports [6, 7].

A linear relationship showing that an increasing number of concurrent multiple risk factors is associated with an increased likelihood of having sICH was found. These findings suggest that increased monitoring is needed in order to reduce the risk of sICH after IVT for AIS patients in northern China.

Our study was conducted at multicenter hospitals, which including secondary hospitals and tertiary hospitals, and this is the greatest advantage of this study. Considering the research hospitals were located in rural and urban areas in northern China, care should be taken in comparing these results with those of previous studies because regional and racial characteristics may act as sources of bias.

This study has some limitations. First, as a retrospective study, the data may have generated system biases. Involved centers may have followed different protocols for IVT. Second,pretreatment Alberta Stroke Program Early CT Score (ASPECTS), hyperdense middle cerebral artery signs and other important parameters that may influence the risk of sICH, were not assessed in the present study. Third, the sample size was relatively small, and the obtained findings should be verified in future studies with larger samples.

## Conclusions

We explored the independent predictive factors as for the risk of sICH within 6 h after IVT in northern China. Patients with the following 3 predictive factors should be closely monitored: an NIHSS score on admission higher than 10, a DNT > 50 min, and a white blood cell count ≥9000/mm^3^.

## Data Availability

All relevant data are within the manuscript. The dataset generated and analyzed in the current study is not publicly available due to privacy constraints relating to the ethical approval and informed consent signed by the participants. Data sharing was not stated in the informed consent signed by the participants.
